# Numerical investigation of soil plugging effect inside sleeve of cast-in-place piles driven by vibratory hammers in clays

**DOI:** 10.1186/s40064-016-2423-y

**Published:** 2016-06-17

**Authors:** Yong Jie Xiao, Fu Quan Chen, Yi Zhi Dong

**Affiliations:** College of Civil Engineering, Fuzhou University, Fuzhou, 350116 China

**Keywords:** Sleeve of cast-in-place pile, Vibratory driving, Soil plugging effect, Finite element method

## Abstract

During driving sleeve of cast-in-place piles by vibratory hammers, soils were squeezed into sleeve and then soil plugging was formed. The physic-mechanical properties of the soil plug have direct influence on the load transmission between the sleeve wall and soil plug. Nevertheless, the researches on this issue are insufficient. In this study, finite element and infinite element coupling model was introduced, through the commercial code ABAQUS, to simulate the full penetration process of the sleeve driven from the ground surface to the desired depth by applying vibratory hammers. The research results indicated that the cyclic shearing action decreases both in soil shear strength and in granular cementation force when the sleeve is driven by vibratory hammers, which leads to a partially plugged mode of the soil plug inside the sleeve. Accordingly, the penetration resistance of sleeve driven by vibratory hammers is the smallest compared to those by other installation methods. When driving the sleeve, the annular soil arches forming in the soil plug at sleeve end induce a significant rise in the internal shaft resistance. Moreover, the influence of vibration frequencies, sleeve diameters, and soil layer properties on the soil plug was investigated in detail, and at the same time improved formulas were brought forward to describe the soil plug resistance inside vibratory driven sleeve.

## Background

It is well-known that the installation of pipe piles may lead to soil plugging. However the existence of the soil plug leads to an increase in vertical bearing capacity and the penetration resistance as well (Gavin and Lehane 2003).

The phenomenon is well-known, but little is known about the mechanism of soil plug formation inside pipe piles, especially for the sleeve driven by vibratory hammers. The formation of a soil plug depends upon a number of factors, e.g. installation method, pile diameter, penetration depth, soil density, whereas the investigation on the influencing factors are still not complete. A lot of researches were conducted to evaluate the soil plugging effect during pipe pile driving. Based on the principle of static equilibrium, Randolph et al. (1991) developed the concept of active height of soil plug and established the one-dimensional static equilibrium equation. Holeyman et al. (2013) presented another analytic expression of the shaft resistance of vibratory driven pile taking account of the soil damping. The limitation of the 1 g model test (O’Neill and Raines 1991) is the same with that of the soil samples under low stress, therefore, the ideal stress condition required by the investigation of the soil plug can be achieved by the use of the geotechnical centrifuge. Nicola and Randolph (1997) analyzed the behavior of soil plug during pipe pile driving by model tests. It was found that the height of soil plug decreased with the increase in the relative compactness of the sands during pile jacking, but increased with the relative compactness of the sands during impact pile driving. Experimental studies of Xing et al. (2012) indicated that the height of soil plug inside impact-driven pipe pile increased as the pipe wall thickness decreased. The distribution of the shaft resistance was nonlinear along the pile length, and depended on the impact force and soil conditions. Tan and Lin (2013) found that the soil plug developed from partially plugged mode in the upper soft cohesive soils into fully plugged mode in the underlying medium dense to dense cohesionless soil layers, and the external shaft resistance was about two-thirds of shaft resistance through a series of steel pipe pile static penetration tests. Liyanapathirana et al. (2000) analyzed the behavior of the soil plug during impact pile driving with Eulerian finite element method, and their researches showed that the degree of soil plug inside the sleeve developed from unplugged mode to partially plugged mode. Thongmunee et al. (2011) analyzed the mechanism and influence of soil plugging bearing capacity performance by discrete element method, and their simulated results were consistent with the experimentation results.

Most of the researches of soil plugging effect focused on the soil plugging mechanisms of jacked or impact-driven pipe piles, instead of vibro-driven pipe piles. However, the difference between the sleeve of cast-in-place piles and pipe piles is obvious, especially when the sleeve diameter is larger or the sleeve wall thickness is thinner, which is vital to the performance of soil plug (Lehane and Gavin 2001; Randolph 2003). The vibratory sleeve driving is a complex mechanical process with respect to the interaction between the sleeve wall and the soils. It involves strong disturbance of the surrounding soils induced by vibration and extrusion, as well as the cyclic shearing and sliding between the sleeve wall and soils. In recent years, some researches on soil plugging effect during vibratory pipe piles driving were conducted according to Henke and Grabe (2008); however, these researches only focused on the soil plugging effect based on the analysis of the radial stress of the soils inside and outside the pile wall. Therefore, more researches with respect to the degree of soil plug, arching effect and influence should be investigated.

## Methods

In this paper, the commercial code ABAQUS was used to simulate the sleeve penetration process in all analyses. An explicit solution algorithm was chosen because it is suitable for problems with large deformations and complex contact conditions. The degree of soil plug and arching effect during vibratory sleeve driving were investigated in the following. At the same time, the influence of vibratory frequencies, sleeve diameters and soil layer properties on the soil plugging was discussed at length, and improved formulas of the soil plug resistance were presented.

### Numerical model

**Fig. 1:**
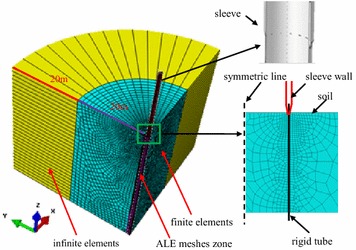
Scheme of FEM meshes and modelling technique

The vibratory sleeve driving into soils is simulated using the commercial code Abaqus/Explicit (Hibbitt et al. 2012). The sleeve length *L* = 20 m, external sleeve diameter *D* = 1 m, and the sleeve wall thickness *T* = 2 cm. The soils directly around the sleeve are discretized by using three-dimensional solid elements with reduced integration. The far field is modeled by infinite elements to avoid wave reflections at the boundary. The sleeve itself is modeled as a rigid body. The finite element mesh scheme used in the study is shown in Fig. 1.

The soils around the sleeve are extruded due to vibratory driving, and the severe distortion of finite element mesh in soils is induced by the continuous cyclic shearing and sliding between the soils and side wall of sleeve. To perfectly solve the problem, the Arbitrary Lagrangian–Eulerian (ALE) adaptive meshing is used in the soil elements within 1 m around the sleeve (Khoubani and Ahmadi 2012).

### Modelling technique for sleeve penetration

The numerical modelling of pile penetration into stiff clays, where the pile was pre-drilled to a depth of 18 m and only the last hammer blows were simulated,was presented by Mabsout and Tassoulas (1994). Soon afterward, a special finite element analysis technique called “zipper-type” was successfully used to simulate the pile installation process. In this modelling technique, a reserved hole with a diameter of 1 % of the pile diameter was simulated in the axis of penetration. This reserve hole was able to expand so that the contact between the soils and pile was established in the penetration process of sleeve. To analyze the soil plugging effect in the open-end pipe pile, Henke and Grabe (2008) developed the numerical modelling of driving open-end pipe pile into soils using the “zipper-type” technique. In their model, a rigid tube of 1 mm wall thickness was created along the open-end pipe pile wall, it penetrated throughout the whole soil layer. Before penetration of pile, the interaction between the pipe pile and soils was assumed to be frictionless. During pile penetration, the pipe pile slided over the rigid tube, and separated the soils from rigid tube. By taking this method, the contact between the driven pile and surrounding soils could be established. The Coulomb friction model was used throughout all analyses for the contact. Due to the full penetration of the rigid tube, the soils at the bottom of the pipe pile couldn’t plug into the pipe pile, which produced a certain influence on the result of soil plugging. This paper simulates the vibratory driving of sleeve, and a rigid tube with a thickness of 1 mm is modelled along the sleeve wall to allow penetration of sleeve (Fig. 1). However, the length of the rigid tube in this model is the same as that of the sleeve, which does not penetrate throughout the whole layer. As the sleeve is being driven, the soils at the bottom of the rigid tube can plug into the sleeve. Therefore, numerical modelling can provide a more precise simulation of the soil plug.

### Load and calculating parameters

The simulation of vibratory sleeve driving in clays is carried out with force control, where the static load *F*_0_ = 85.5 kN, vibration frequency *f* = 25 Hz, dynamic loading amplitude $F_{\text{c}} = 2600$ kN and exciting force $F_{\text{d}} = F_{0} + F_{\text{c}} \sin (2\pi ft)$ are applied to the sleeve.

The mechanical behavior of soils is described by Mohr–Coulomb constitutive model. Since the total stress analysis is adopted in the Abaqus/Explicit, the undrained strength parameters for soil layers obtained by the UU tests are used (Table 1). The generalized Gibson soil (Gibson 1967), where the soil modulus linearly increases with the depth, is used as follows

1 $$E_{\text{z}} = E_{0} + nz$$

where *z* is the soil depth below the ground surface, *E*_z_ is the soil modulus at the depth *z*, *E*_0_ is the soil modulus at the ground surface, *n* is the Gibson’s soil parameter.

**Table 1:** Parameters of clays

Soil	*γ*/(kN/m^3^)	*E*_0_/(MPa)	*ν*	*c*_u_/(kPa)	$\varphi_{\text{u}}$ /(degree)
Clay	18	10	0.49	28	0

As for the friction angle of the interface between pile and soils *δ*, Potyondy’s (1961) research indicated that value of $\delta /\varphi^{\prime}$ between 0.6 and 0.7 is more suitable for the clays. The effective internal friction angle $\varphi^{\prime}$ = 12°–32°, and *δ* = 12°–22°. Thus, the friction coefficient *μ* = tan *δ* is in the range of 0.2–0.4. Friction coefficient *μ* was set to be 0.25 in this paper.

The transmission process of vibration waves is influenced by the value of the material damping. The lower the damping is, the slower the waves attenuate. ABAQUS provides Rayleigh damping including two damping parameters. The mass proportional damping $\alpha_{\text{R}}$ is a proportional coefficient about the mass matrix, and it is mainly used in calculating the damping force induced by absolute motion of model and simulating the model movement in the static viscous fluid. The stiffness proportional damping $\beta_{\text{R}}$ is a proportional coefficient about the stiffness matrix, and it is mainly used in calculating the damping force induced by soil strain rate. There is no absolute motion in the soil model due to the boundary constraint. Thus, only the stiffness proportional damping is needed to be introduced in the finite element model. The equation of stiffness-proportional damping $\beta_{\text{R}}$ is given by

2 $$\beta_{\text{R}} = 2\xi /\omega_{1}$$

where *ξ* is the damping coefficient, and *ω*_1_ is the first natural frequency.

The natural frequency extraction analysis of the soil model can be carried out by Abaqus/Standard, and the obtained first natural frequency is 1.28 Hz. The damping coefficient *ξ* used in this paper is the recommended value of 2 % by Ekanayake et al. (2013). From Eq. (2), the stiffness proportional damping $\beta_{\text{R}}$ is 0.031.

## Results and discussion

### Void ratio variation

Because Abaqus/Explicit can’t output the void ratio datum, the void ratio can only be achieved by the strain-void ratio relationship, in order to investigate the rule of void ratio change during sleeve driving.

According to the elastic–plastic theory, the volumetric strain $\varepsilon_{\text{v}}$ of the soils can be achieved by the following function

3 $$\varepsilon_{\text{v}} = \varepsilon_{\text{x}} + \varepsilon_{\text{y}} + \varepsilon_{\text{z}}$$

When the void ratio changes from *e*_1_ to *e*_2_, the corresponding volumetric strain is (Das 2014)

4 $$\varepsilon_{\text{v}} = \frac{{e_{1} - e_{2} }}{{1 + e_{1} }}$$

From the above equations, the void ratio increment can be given by

5 $$\Delta e = - (1 + e_{0} )(\varepsilon_{\text{x}} + \varepsilon_{\text{y}} + \varepsilon_{\text{z}} )$$

where the Δ*e* is the void ratio increment, Δ*e* = *e* − *e*_0_, *e* is corresponding void ratio (at the time t), *e*_0_ is the initial void ratio. If the Δ*e* were positive, the void ratio would be larger than the initial void ratio, which means soils become loose. If the Δ*e* were negative, the void ratio would be smaller than the initial void ratio, which indicates the soils are compacted.

**Fig. 2:**
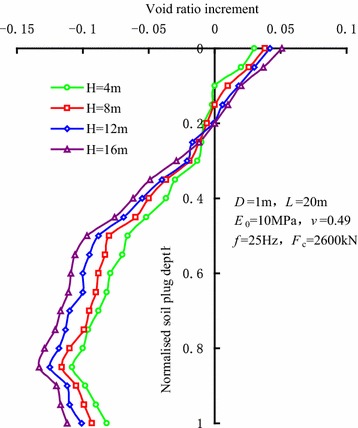
Variations of void ratio increment of soils in the centre of the sleeve

Figure 2 shows the variations of void ratio increment of soils in the centre of the sleeve during high-frequency vibratory sleeve driving. In this figure, *H* is the penetration depth. For the normalized soil plug height of this figure, 0 is the top of the soil plug, and 1 is the bottom of the soil plug. The figure indicates that as the penetration depth of the sleeve increases, the void ratio of the soils at the bottom of soil plug decreases as the soils are extruded to a dense state, while the disturbance of soils at the top of the soil plug gives rise to an increase in void ratio as the soils are loosened. The analysis implies that the soil plug at the bottom undergoes not only the continuous cyclic shear from the sleeve, but also the extrusion from the soils around the sleeve end and the pressure from the soils above the soil plug. Consequently, the soil density increases, which leads to a decrease in void ratio. However, due to the fact that the upper part of soil plug is not constrained, the effect of disturbance from the sleeve wall on the upper part of soil plug results in an increase in void ratio.

### Internal shaft resistance

**Fig. 3:**
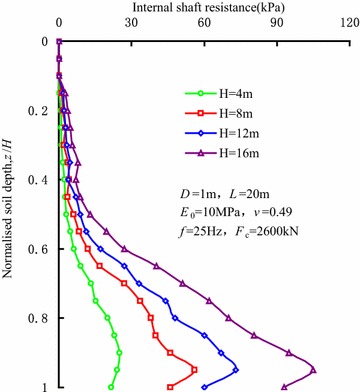
Distributions of internal shaft resistance during vibratory sleeve driving

The distributions of internal shaft resistance along the sleeve wall during vibratory driving are shown in Fig. 3. It can be seen that the sliding deformation occurs between the sleeve wall and the bottom of soil plug, and the internal shaft resistance reaches the maximum value. On the contrary, the internal shaft resistance at the soil plug top is close to 0.

The internal shaft resistance tends to increase with the penetration depth, and depends on the friction coefficient between the soils and sleeve wall and the normal stress applied on the sleeve wall. As the penetration depth increases, the radial stress in the initial stress state of soil plug becomes larger, and the arching effect at the bottom of the soil plug is more obvious. This is because the lateral deformation is constrained by the relative rigid sleeve, which induces an increase in horizontal stress and internal shaft resistance.

### Degree of soil plug

In the vibratory sleeve penetration process, there are three degrees of soil plug inside the sleeve, namely the unplugged, partially plugged and fully plugged. When the soil plug is fully plugged, the soil plug doesn’t slide along the sleeve wall. Hence, the open-end piles in fully plugged mode behave like closed-end piles. Nevertheless, there is relative sliding between the soil plug and sleeve wall in other types of plugged mode.

**Fig. 4:**
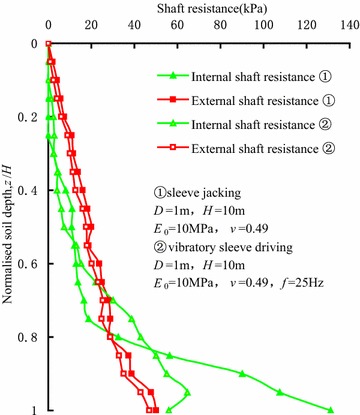
Distributions of shaft resistance along the sleeve varying with the normalized soil depth

Figure 4 shows the distributions of shaft resistance along the sleeve varying with the normalized soil depth, namely the ordinate is *z*/*H*. The internal shaft resistance at the bottom of vibro-driven sleeves is clearly smaller than that of jacked sleeves. Therefore, the possibility of the development of fully plugged mode inside sleeve during vibratory driving (Henke and Grabe 2008) is low. The effective length, where the internal shaft resistance is bigger than external shaft resistance, of vibro-driven sleeve is longer than that of jacked sleeve. This means that the effective length of internal shaft resistance is evidently influenced by the degree of soil plug. Further research results show that the penetration resistance of the sleeve during vibratory driving is smaller than that during jacking.

The degree of soil plug can be judged by the incremental filling ratio (*IFR*) (Paik and Rodrigo 2003), and the incremental filling ratio can be determined by

6 $$IFR = \frac{{{\text{d}}h}}{{{\text{d}}H}} \times 100\;\%$$

where *h* is the average height of soil plug, *H* is the penetration depth. The unplugged and fully plugged mode correspond to *IFR* = 100 % and *IFR* = 0, respectively. A value of *IFR* varying from 0 to 100 % indicates that the soil plug is partially-plugged.

**Fig. 5:**
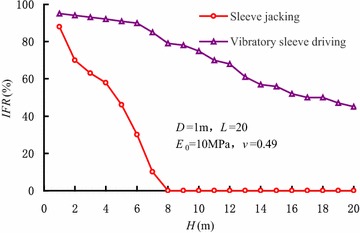
Variations of *IFR* during sleeve driving

Figure 5 shows the variations of *IFR* during sleeve driving. When the sleeve is driven into a shallow soil layer, the value of *IFR* is relatively larger, which indicates a rapid increase in the height of soil plug. As the penetration depth increases, the *IFR* shows a tendency to decrease during sleeve jacking. When the sleeve reaches the depth of 8 m, the soil plug inside the sleeve becomes fully plugged. On the contrary, *IFR* decreases gradually with an increase in the degree of soil plug when the sleeve is driven by vibratory hammers, and the soil plug is partially-plugged during whole penetration process.

When the sleeve is being driven, the sleeve transfers the periodic vibrating load to the soils around the sleeve (Ekanayake et al. 2013). The strain in the soils around the sleeve continues to increase, and the cohesion and shear strength gradually decrease. Compared to the infinite soils outside the sleeve, the soil plug inside the sleeve undergoes stronger cyclic shear force transferred from the sleeve wall. Therefore, the fluctuation of the internal shaft resistance is more obvious than that of the external shaft resistance. As the result, the soil strength and cohesion decrease under cyclic shearing action. Because of the decrease in the soil strength and cohesion, the soil plug becomes partially-plugged, and the penetration resistance of sleeve is much smaller than those by other installation methods.

### Arching effect

**Fig. 6:**
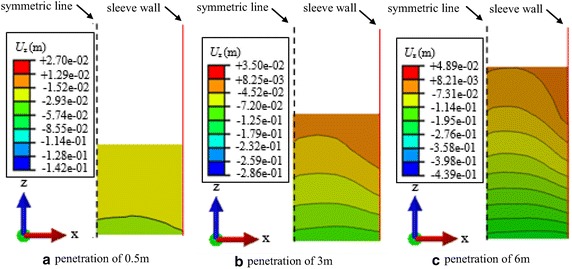
Vertical displacement isolines of the bottom of soil plug and corresponding legend. **a** Vertical displacement isolines of the bottom of the soil plug at penetration depth of 0.5 m. **b** Vertical displacement isolines of the bottom of the soil plug at penetration depth of 3 m. **c** Vertical displacement isolines of the bottom of the soil plug at penetration depth of 6 m

Figure 6 shows the vertical displacement isolines of the bottom of soil plug at different penetration depths. Since the shaft resistance is small when the sleeve penetrates into a shallow soil layer, no obvious arching effect occurs at the bottom of soil plug during the early stage of penetration. Therefore, the shaft resistance and shear displacements occur only in the soils around the sleeve. However, there is no obvious displacement of the soils in the interior of sleeve comparing to the displacement of sleeve. The internal shaft resistance increases along with the penetration depth of the sleeve, which results in the large settlement of soil plug around the sleeve wall. Therefore, arched displacement isolines occur, which represents that the soil plug upheaval in the middle of the sleeve is not the largest. While Nicola and Randolph (1997) found that a convex arch was formed in the soil plug of the pile end during pipe pile jacking. The difference was that the soils around the sleeve wall were towed downward along with the sleeve during vibratory driving. When the sleeve was pulled out, the soils were towed upward by the sleeve wall as well. Therefore, the soils in the interior of the sleeve were slightly influenced by the towing effect of the sleeve wall, and settled due to vibratory driving sleeve, where the upheaval was relative small compared to the soils around the sleeve. Further research results show that a convex arch is formed in the soil plug of sleeve end, and tends to be flatter as the sleeve diameter increases, since the arching effect is weakened as the increase in sleeve diameter.

Figure 6 also shows that the vertical displacements at the bottom of the soil plug increase with the penetration depth, which also indicates the arching effect becomes stronger as the penetration depth increases.

**Fig. 7:**
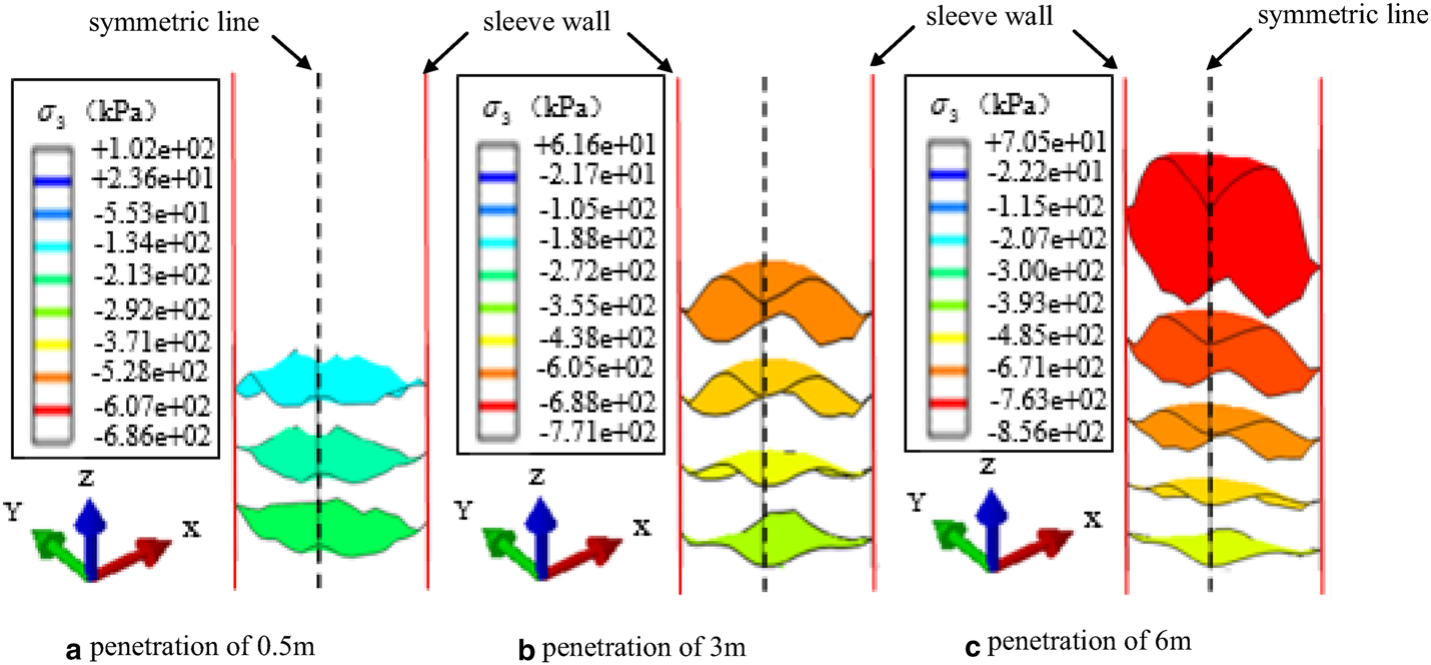
Principal stress isosurfaces of the bottom of the soil plug and corresponding legend. **a** Principal stress isosurfaces of the bottom of the soil plug at penetration depth of 0.5 m. **b** Principal stress isosurfaces of the bottom of the soil plug at penetration depth of 3 m. **c** Principal stress isosurfaces of the bottom of the soil plug at penetration depth of 6 m

In ABAQUS, the tensile stress is assumed to be positive. Figure 7 shows the principal stress isosurfaces of the bottom of the soil plug at different penetration depths, and indicates that stress at the bottom of soil plug is compressive stress. It can be seen that at the early stage of penetration, the principal stress isosurfaces basically take on a horizontal distribution, and no obvious arching effect occurs. As the sleeve is driving down, the soil particles move downward along with the sleeve and the lower soils become denser. Due to extrusion from the soils at the sleeve bottom and shaft resistance from the sleeve wall, the ring stress arches are formed in the lower part of the soils, of which the phenomenon is induced by the large internal shaft resistance against the sleeve wall, when the soils are squeezed into the sleeve.

Figure 7 also shows the stress arch is most obvious within a certain range from the sleeve end. Within the range, the soil plug undertakes the major part of the internal shaft resistance, and the stress value increases with the penetration depth. The simulation results accord with the study of Lobo-Guerrero and Vallejo (2007), the contact stress mainly was concentrated at the bottom of sleeve end, and was delivered to the interior of the sleeve in a short distance from the sleeve end. During sleeve jacking, the void ratio is slightly smaller than the void ratio in initial stress state due to the monotonic shear between the soils and sleeve wall. Compared to the sleeve jacking, the arching effect of vibratory sleeve driving is relatively weaker, since the cyclic shear between the soils and sleeve wall makes void ratio decrease to the minimum value (Henke and Grabe 2008). The soil plug is compacted under vibratory action, which makes the soils at the bottom of sleeve difficult to be squeezed into the sleeve. Consequently, the arching effect is relatively weaker.

### Sensitivity analysis

#### Vibration frequency

**Fig. 8:**
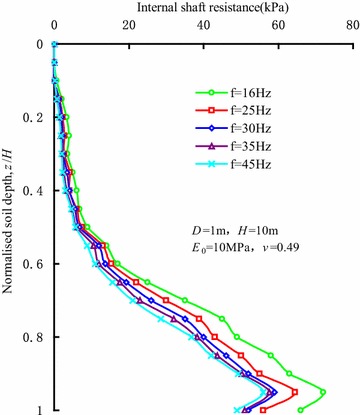
Distributions of internal shaft resistance along the sleeve varying with vibration frequencies

Figure 8 shows the distributions of internal shaft resistance along the sleeve varying with vibration frequencies at the penetration depth of 10 m. The research results show that a threshold value exists in the influence of the vibration frequency on the internal shaft resistance. When the vibration frequency is less than 30 Hz, the internal shaft resistance will decrease with the increase in the vibration frequency. When the vibration frequency reaches 30 Hz, the influence of vibration frequency is not obvious on internal shaft resistance. Rodger and Littlejohn (1980) also proved the existence of the threshold value in the vibration frequency through experiments. The stress waves induced by vibration reduce the contact pressure, which gives rise to the reductions in the friction strength in the granular soils and the shaft resistance between the soils and the sleeve wall. When the vibration frequency reaches a certain value, the shaft resistance between the soils and sleeve wall decreases significantly and even disappears.

**Fig. 9:**
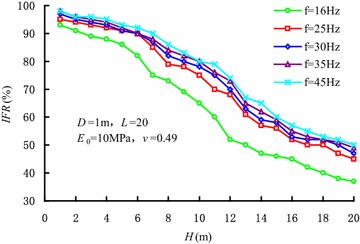
Variations of *IFR* with penetration depth varying with vibration frequencies

Figure 9 shows the variations of *IFR* with penetration depth varying with vibration frequencies. It can be seen that *IFR* is always over 0 and the soil plug is partially- plugged. A threshold value exists in the influence of vibration frequency on the *IFR* as well. When the vibration frequency is less than 30 Hz, the *IFR* increases with the increase in vibration frequency, and the degree of soil plug becomes weaker accordingly. When the vibration frequency reaches 30 Hz, the influence of vibration frequency isn’t obvious. At the same time, the degree of soil plug is slightly influenced by vibration frequency.

#### Sleeve diameter

**Fig. 10:**
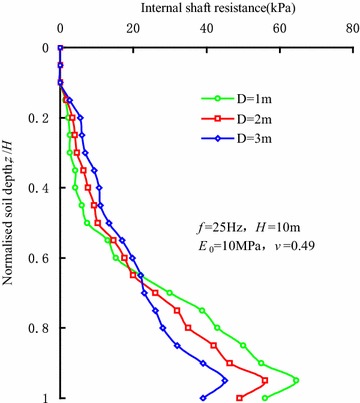
Distributions of internal shaft resistance along the sleeves of different sleeve diameters

Figure 10 shows the distributions of internal shaft resistance along the sleeves of different sleeve diameters at the penetration depth of 10 m. It can be seen that the internal shaft resistance decreases with the increase in sleeve diameter in the range of 0.3*H* to 0.4*H* from the sleeve end. Beyond this range, the internal shaft resistance increases with the increase in sleeve diameter. This trend is also observed for different penetration depths and vibration frequencies. Based on the analysis results, the increase in the sleeve diameter weakens the constraint at the bottom of the soil plug from the sleeve wall, thus the decreasing arching effect leads to a reduction in the internal shaft resistance as well. Moreover, the cohesion strength and shear resistance in the upper part of soil plug decrease due to the vibrating load. Compared to the infinite soils outside the sleeve, the soil plug inside the sleeve undertakes stronger cyclic shear from the sleeve wall such that the internal shaft resistance is smaller than the external shaft resistance. When the sleeve diameter tends to be large enough, the boundary condition of soil plug is transferred from one-dimension soil column to the unconfined boundary. Therefore, the internal shaft resistance of the upper part of the sleeve increases gradually with the increase in sleeve diameter, and is close to external shaft resistance.

**Fig. 11:**
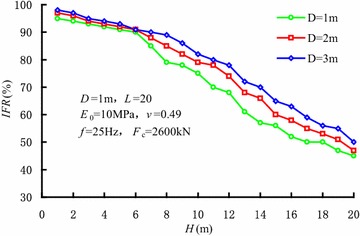
Variations of *IFR* varying with penetration depth of different sleeve diameters

Figure 11 shows the variations of *IFR* varying with penetration depth of different sleeve diameters. The soil plug is partially-plugged during vibratory driving. *IFR* increases gradually as the sleeve diameter increases, while the degree of soil plug decreases as the sleeve diameter increases.

#### Soil layer

**Fig. 12:**
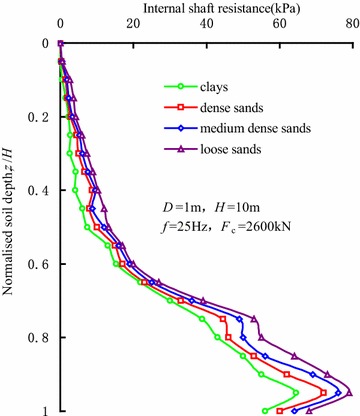
Distributions of the internal shaft resistance along sleeve in different soil layers

Figure 12 represents the distributions of the internal shaft resistance along sleeve in different soil layers at the penetration depth of 10 m, and the sand parameters are shown in Table 2. The influence of soil condition on the internal shaft resistance is clear from the figure. Furthermore, the internal shaft resistance in clays is relatively smaller than that in sands. Because of the soil structure of clays, the disturbance incurred by high-frequency driving into the clays is more obvious than that in the sands.

**Table 2:** Parameters of sandy soils

Density	*γ*/(kN/m^3^)	*E*_0_/(MPa)	*ν*	*c*_u_/(kPa)	$\varphi_{\text{u}}$/(degree)
Dense	20	40	0.25	0	40
Medium dense	19	30	0.27	0	36
Loose	18	25	0.30	0	30

Figure 12 also indicates that the internal shaft resistance inside the sleeve decreases as the sand compactness increases. Vibratory driving can effectively turn the loose sands inside the sleeve into medium dense sands, which leads to an increase in the internal shaft resistance. The dilatancy effect is also induced by vibratory driving in the dense sands, which indicates that the void ratio increases. As the result, the internal shaft resistance decreases correspondingly.

### Analytic solution of soil plug resistance

#### Active height of soil plug

**Fig. 13:**
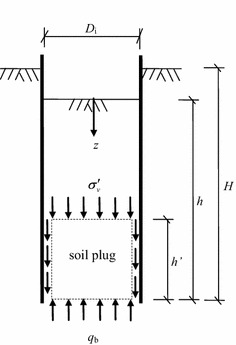
Active height of soil plug after Randolph et al. (1991)

Based on the silo theory, Randolph et al. (1991) proposed the concept of the active height $h^{\prime}$ of soil plug. It is assumed that the soil plug inside the pipe pile moves up relatively to the pipe pile wall during installation, the soils within the active height of soil plug undertake the major part of internal shaft resistance of pipe pile, see Fig. 13. In this paper, the numerical study shows that the soil plug become partially-plugged during vibratory driving, in which case the soil plug moves upward relatively to the sleeve wall. The stress arches within a certain distance from the sleeve end are most obvious, and the soil plug in this zone undertakes the major part of the internal shaft resistance. As a result, the soil plug inside the sleeve during vibratory driving is consistent with Randolph’s assumption, so the active height of soil plug is the height of soil arch.

**Fig. 14:**
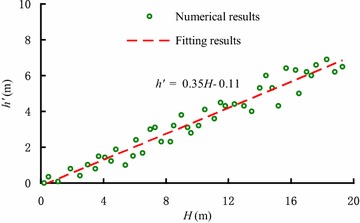
Relationship between penetration depth and active height of soil plug

According to the numerical simulation, the results of the penetration depth and active height of the soil plug under various conditions, namely different vibration frequencies and sleeve diameters, shows that the relationship between *H* and *h*^′^, which can be seen in the Fig. 14, can be described by the following linear regression function

7 $$h^{\prime} = 0.35H - 0.11$$

Figure 14 shows there is no arching effect at the early stage of sleeve penetration, the active height of soil plug is 0. As the penetration depth increases, the soil plug moves down constantly, which gives rise to the arching effect. The relationship between the active height of soil plug and penetration depth is basically linear.

#### Soil plug resistance

**Fig. 15:**
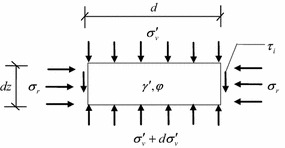
Forces acting on an infinitesimal disc of the soil plug

The soil plug resistance has been explored in detail by Randolph et al. (1991), who showed that the soil plug resistance increases exponentially with soil plug length. In Fig. 15, it can be found that the soil plug can be simplified as a series of horizontal discs; the forces acting on the infinitesimal disc are the effective vertical stress $\sigma^{\prime}_{\text{v}}$ at the top and bottom of the soil discs, the shear stress $\tau_{\text{i}}$ along the sleeve wall, and the soil effective unit weight $\gamma^{\prime}$.

The equation of vertical equilibrium of the soil disc of soils at any depth *z* can be given by

8 $$\frac{{{\text{d}}\sigma^{\prime}_{\text{v}} }}{{{\text{d}}z}} = \gamma^{\prime} + \frac{4}{{D_{\text{i}} }}\tau_{\text{i}}$$

The internal shear stress between the soils and the sleeve wall can be determined by

9 $$\tau_{\text{i}} = \beta \sigma^{\prime}_{\text{v}}$$

where $D_{\text{i}}$ is the internal sleeve diameter, *β* is the coefficient depending on the earth pressure coefficient *K* and contact friction coefficient tan *δ*, and equation of *β* is given below

10 $$\beta = \frac{{\sin \varphi^{\prime}\sin (\Delta - \delta )}}{{1 + \sin \varphi^{\prime}\cos (\Delta - \delta )}}$$

11 $$\sin \Delta = \frac{\sin \delta }{{\sin \varphi^{\prime}}}$$

where $\varphi^{\prime}$ is the effective internal friction angle, *δ* is the friction angle between sleeve wall and soils.

The assumed boundary pressure *p* is placed on the top of the soil plug, and the integral of the Eq. (8) yields the effective vertical stress at any depth z of soil plug as follows

12 $$\sigma^{\prime}_{\text{v}} = p + \left( {e^{{4\beta z/D_{\text{i}} }} - 1} \right)\left( {p + \frac{{\gamma^{\prime}D_{\text{i}} }}{4\beta }} \right)$$

The initial stress of at the bottom of soil plug is *p* + *γ*^′^*h*, therefore the additional stress at the bottom of soil plug can be described by

13 $$q_{\text{b}} = \left( {e^{{4\beta h/D_{\text{i}} }} - 1} \right)\left( {p + \frac{{\gamma^{\prime}D_{\text{i}} }}{4\beta }} \right) - \gamma^{\prime}h$$

From the Eq. (12) to (13), the total internal shaft resistance $Q_{\text{si}}$ and soil plug end resistance $Q_{\text{p}}$ can be described by Eqs. (14) and (15) respectively, as follows

14 $$Q_{\text{si}} = \pi D_{\text{i}} \int\limits_{0}^{h} {\beta \sigma^{\prime}_{\text{v}} {\text{d}}z}$$

15 $$Q_{\text{p}} = \frac{{\pi D_{\text{i}}^{ 2} }}{4}q_{\text{b}}$$

When the degree of soil plug is fully-plugged, total internal shaft resistance $Q_{\text{si}}$ is larger than soil plug end resistance $Q_{\text{p}}$, and soil plug resistance $Q_{\text{plug}}$ is equal to soil plug end resistance $Q_{\text{p}}$. When the degree of soil plug is partially-plugged, total internal shaft resistance $Q_{\text{si}}$ is smaller than soil plug end resistance $Q_{\text{p}}$, and soil plug resistance $Q_{\text{plug}}$ is equal to total internal shaft resistance $Q_{\text{si}}$.

When the soil plug is partially-plugged during vibratory driving, the soil plug resistance $Q_{\text{plug}}$ is equal to the total internal shaft resistance $Q_{\text{si}}$. According to the theory of active height of soil plug of Randolph et al. (1991), the internal shaft resistance is mainly undertaken by the soils within the active height of soil plug. While the soil plug beyond the active height of soil plug contributes little in withstanding internal shaft resistance, the increase in soil plug beyond the active height of soil plug can be ignored. As a result, the internal shaft resistance can be simplified as the shaft resistance within the active height of soil plug, so the average height of soil plug *h* can be taken as the penetration depth *H*. From the Eq. (12) to (14), the soil plug resistance during vibratory driving can be described by

16 $$Q_{\text{plug}} = \pi D_{\text{i}} \int\limits_{{(H - h^{\prime})}}^{H} {\beta \left[ {p + \left( {e^{{4\beta z/D_{\text{i}} }} - 1} \right)\left( {p + \frac{{\gamma^{\prime}D_{\text{i}} }}{4\beta }} \right)} \right]} {\text{d}}z$$

**Fig. 16:**
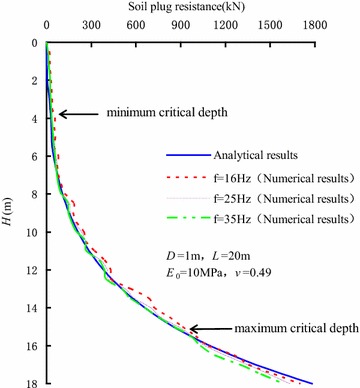
Variations of soil plug resistance with penetration depth

Figure 16 describes the variations of soil plug resistance with penetration depth. The analytic solution of soil plug resistance in the figure can be obtained from the Eq. (16). However, the numerical solution of soil plug resistance is obtained through the integral of shear stress between the sleeve wall and soil contact surface along the sleeve. The soil plug resistance exhibits exponential distribution along the penetration depth, which is consistent with the research conducted by Igoe et al. (2011).

As shown in Fig. 16, the value of soil plug resistance of numerical solution is slightly greater than that of analytic solution at the early stage of sleeve driving. When the sleeve reaches a certain depth, the numerical solution agrees well with analytic solution, and this certain depth is defined as minimum critical depth. Because the arching effect is weak during the penetration of sleeve, the soil plug resistance is withstood by the whole soil plug. As a result, the numerical solution obtained from the integral along the sleeve length is larger than the analytic solution obtained from the integral along the active height of soil plug. When the sleeve is driven into deep soil layers, an obvious arching effect occurs in soil plug. At this time, the soils within the active height of soil plug undertake the major part of the soil plug resistance, and soil plug resistance obtained from the numerical simulation is identical to that given by analytic study. As the penetration depth increases, a maximum critical depth exists in the penetration process. When the penetration depth is deeper than the maximum critical depth, the numerical solution of soil plug resistance is smaller than analytic solution. This phenomenon can be explained by the fact that the cyclic shearing and sliding between the sleeve wall and deep soils lead to plastic failure in soils.

Figure 16 also shows that when *f* < 25 Hz, the numerical solution is slightly different from the analytic solution. When *f* ≥ 25 Hz, the numerical solution is identical to the analytic solution relatively. Distinctly, when *f* ≥ 25 Hz, a more precise solution of soil plug resistance can be obtained by Eq. (16). The regression analysis indicates that the minimum critical depth is 3.2*D*, and the maximum critical depth is 15.5*D*. Therefore, the soil plug resistance obtained from the Eq. (16) is proved to be reasonable, if it satisfies the condition *f* ≥ 25 Hz and 3.2*D* < *H* < 15.5*D*.

## Conclusions

According to the coupled finite-infinite element modelling of sleeve penetration by vibratory hammers, the soil plugging effect during the penetration was investigated. The main conclusions were as followings: (1) During vibratory sleeve driving, the soil strength and cohesion decrease, which is induced by cyclic shearing action. As a result, soil plug is partially-plugged, and the penetration resistance is less than those by other installation methods; (2) In the vibratory penetration process, annular soil arches form inside the soil plug, which induces an obvious increase in internal shaft resistance. The soils within the active height of the soil plug undertake the major part of the internal shaft resistance; (3) A threshold value exists in the influence of the vibration frequency on internal shaft resistance. When the vibration frequency is less than the threshold value, internal shaft resistance decreases with the increase in vibratory frequency, which correspondingly reduces the degree of soil plug. When the vibration frequency reaches the threshold value, the influence of vibration frequency on internal shaft resistance can be ignored. Hence, the vibration frequency has little influence on the degree of soil plug; (4) The internal shaft resistance of the sleeve end gradually decreases as the sleeve diameter increases, while the internal shaft resistance in the upper part of the sleeves increases as the sleeve diameter increases. Moreover, the increase in sleeve diameter gradually decreases the degree of the soil plug. The internal shaft resistance depends closely on the soil layer properties as well; (5) During penetration of the sleeve, the distribution of soil plug resistance along the height of soil plug can be described by index curve. Soil plug resistance is mainly undertaken by the soils within the active height of soil plug. At the same time, the analytic expression of soil plug resistance during vibratory driving was given under the conditions of *f* ≥ 25 Hz and 3.2*D* < *H* < 15.5*D*.
